# Camillo Golgi’s contributions to the anatomic basis of sensitivity in tendons

**DOI:** 10.1007/s00702-024-02826-7

**Published:** 2024-10-26

**Authors:** Maria Carla Garbarino, Antonio Pisani, Marco Biggiogera, Paolo Mazzarello

**Affiliations:** 1 Department of Brain and Behavioral Sciences, University of Pavia, Pavia, Italy; 2 Museum System, University of Pavia, Pavia, Italy; 3 IRCCS Mondino Foundation, Italy Pavia,; 4 Department of Biology and Biotechnology “L. Spallanzani”, University of Pavia, Pavia, Italy

**Keywords:** Camillo Golgi, Vittorio Mazzoni, Golgi muscle-tendon organs, Golgi-Mazzoni corpuscles

## Abstract

Between 1878 and 1880 Camillo Golgi, professor of Histology and General Pathology at the University of Pavia, studied the termination of the nerves inside the tendons, near their muscular insertion. He defined two fundamental categories of corpuscles. The first type, which he called muscle-tendon terminal organs, was morphologically characterized by spindle structures which at one end seemed to relate to the muscle fibers while at the other end they gradually merged with the tendon bundles. Golgi discovered that these structures received from one to four myelinated nerve fibers, which lost their myelin sheath as they entered the bundle, within which they divided dichotically, ending in a large number of terminal arborizations that had the appearance of reticular intertwines. In the superficial thickness of the tendon, near the muscle, Golgi also noticed a second category of corpuscles, which he described as claviform bodies or formations similar to Pacinian bodies. In 1890 Vittorio Mazzoni precisely defined their morphological characteristics. These corpuscles were later called Golgi muscle-tendon organs and Golgi-Mazzoni corpuscles. On the basis of their position and histological appearance, Golgi also correctly hypothesized their physiological role: to be receptors of muscular tension for the muscle-tendon organs and transducers of sensitivity to touch and pressure for the Golgi-Mazzoni corpuscles.

## Introduction

From a conceptual point of view, the problem of sensitivity was one of the oldest among those that physiology posed to philosophy. In the second half of the eighteenth century, in the midst of the Enlightenment, the problem was addressed on an experimental basis by the great Swiss physiologist Albrecht von Haller (1708–1777) who hypothesized the existence of two fundamental characteristics of living matter: irritability and sensitivity (Monti 2002; Piccolino and Bresadola 2013). Thanks to the former, some parts of the living body (muscle bundles) reacted to the stimulation with contractions; thanks to sensitivity, however, the innervated tissues reacted with manifestations of pain or sensitive impressions. Research on perception, however, took a leap forward thanks to the development of microscopic technique, which put the study of perceptual gateways possible, i.e. the structures that allow us to perceive both the external world and the internal state of organs, on the anatomical research agenda. From the mid-19th century onwards, sense organs were interpreted on the basis of cell theory. In this way, it was realized that there are specialized sensory cells, referred to as ‘receptive cells’, specifically sensitive to definite sensory stimuli.

Some of them, linked to very specialized sensory organs, have been named basing on their shape (cones, rods, hair cells, etc.), while those in the skin or under the surface of the skin are generally named after the person who first described them (Wade 2018).

**Fig. 1:**
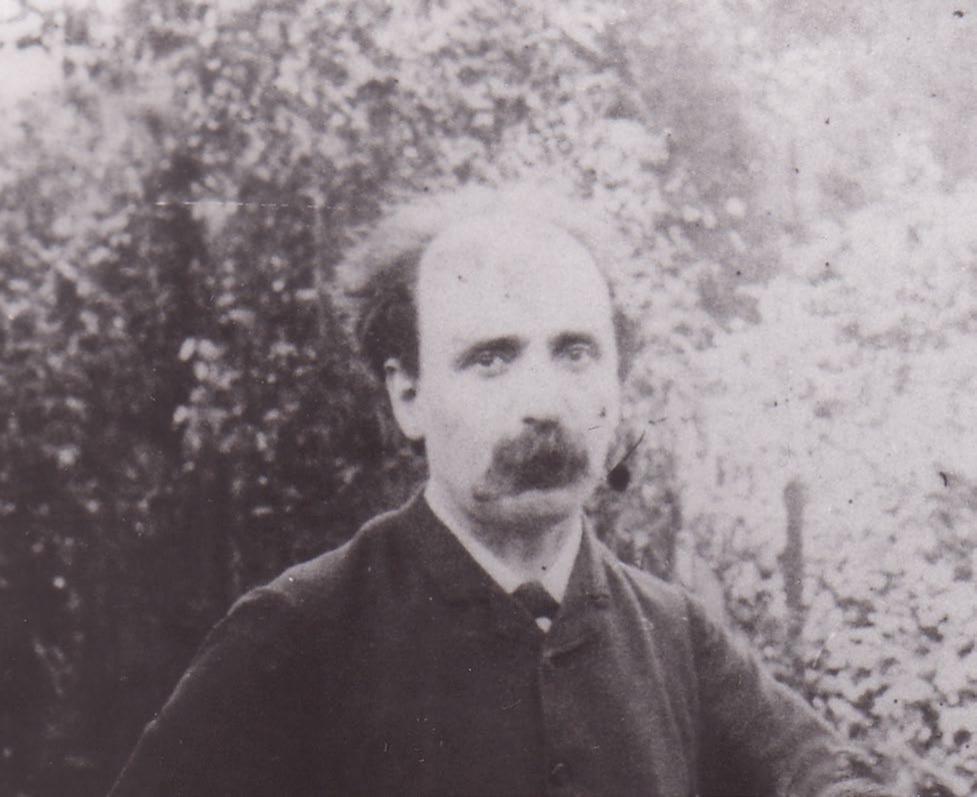
Camillo Golgi around 1877–1878, at the time of his research muscle-tendon organs. University of Pavia, Camillo Golgi Museum

Among the scientists who gave a fundamental contribution to the characterization of sensory corpuscles, Camillo Golgi (1843–1926) deserves an important place. Five years after developing the *black reaction*, with which he succeeded in highlighting the fine architecture of the central nervous system (Mazzarello 2010, 2019), Golgi was able to characterize two important sensory corpuscles located in tendons (Fig. 1).

## Becoming an eponym

In 1880 Vittorio Marchi (1851–1908) proposed calling Golgi’s body a particular structure that Golgi had discovered two years earlier in the thickness of muscle.

Golgi came to this discovery early in his university career, having been appointed to the chair of Histology at the University of Pavia in 1876. The call to Pavia marked the culmination of a long and difficult period in search of a stable university position that would allow him to continue the scientific activity that had enthused him during his years of study at the University of Pavia.

Born in Corteno (today Corteno Golgi) on 7 July 1843, Golgi enrolled at the University of Pavia with the sole intention of obtaining a university degree in medicine in order to work as a practitioner, like his father had done many years before. However, after graduating in 1865, his contacts with already influential scholars such as Cesare Lombroso (1835–1909) and Giulio Bizzozero (1846–1901) converted him to scientific research. After a few years of temporary employment at the University of Pavia, he started working as chief physician in a hospital for chronic patients, the “Pia Casa degli Incurabili” in Abbiategrasso, about thirty kilometers from Pavia.

Working in this hospital, “with the sacred fire of scientific research”, as he wrote later, between 1872 and 1873 he had succeeded in fine-tuning a histological procedure for staining nerve structures (the *black* or *chromo-argent reaction*, also known as *Golgi’s method*), which fulfilled every neuroanatomist’s dream of being able to clearly highlight the basic structure of the central nervous system.

The method consists in an initial phase of ‘fixation’ of the nerve tissue in potassium dichromate, for a time varying from fifteen days to more than a month, followed by a passage in silver nitrate for a minimum time of one or two days (but sometimes the reaction develops already after two or three hours). The obtained result is the random but selective precipitation of a salt, silver chromate, which occupies and colors the cell body and all the extensions to their extreme branches and ramifications, black. For reasons that are still completely unknown, only a small percentage, varying between 1 and 5% of the cells present in the microscopic field, is colored. Thus, the silhouette of the single nerve cell appears in all its complex morphology. So, from the incredible, labyrinthine complexity of the nerve tissue, it is possible to extract the single element, which emerges like a tree, with all its branches and roots, from an inextricable forest. The nervous arabesque, previously unknowable, became decipherable from that moment on. Golgi immediately understood the importance of the extraordinary instrument he had created. By employing this method, a generation of neuroanatomists, among whom the great Spanish scientist Santiago Ramón y Cajal (1852–1934), was able to lay the conceptual foundations of modern neuroscience based on the neuron theory.

Thanks to this fundamental methodological innovation and the many researches that followed, Golgi was awarded the chair of histology and general pathology in Pavia and became one of the leading figures in histological and microscopic anatomy research between the 19th and 20th centuries, gaining important international recognition, including the Nobel Prize for medicine in 1906 (*ex aequo* with Santiago Ramón y Cajal). With a variation of his method, he was also able to describe one of the fundamental constituents of the cell, initially referred to as the *internal reticular apparatus* and later as *the Golgi apparatus* or *the Golgi*.

However, Golgi’s great fame is due not only to the studies he carried out with the black reaction but also to his research on the malaria cycle in human blood (1886–1892), during which he described the development cycle of the plasmodium (the protozoan that causes malaria) in human blood (*Golgi cycle*) and the law of correlation between the multiplication of the parasite and fever access (*Golgi’s law*) (Lambertini 1949).

### Golgi’s laboratory

In 1876, Golgi obtained, in addition to the chair of Histology, a modest space to conduct laboratory activities, located in the Botanical Garden building, equipped with few instruments. However, the narrowness of the space and the poverty of the equipment did not prevent the laboratory from quickly becoming a prestigious research center. A few years later, the laboratory was described by one of Golgi’s students, Antonio Pensa (1874–1970), as follows:

“It consisted of three rooms, in addition to the director’s room, overlooking the Botanical Garden. Golgi’s work room was a cramped space, about three meters wide and no more than four meters long […]. A magnificent disorder reigned there […] On the floor, in the corners and along the walls, jars containing liquids of the most different colors in which various organic elements were stored and barely recognizable: spinal cords or whole brains or cut into pieces. Leaning against one side wall was a cabinet for storing the preparations, and at the other opposite was a signboard and a small glass cabinet, with flasks of various sizes and various colors; in the middle of the room was a table where many papers, books, newspapers were piled up. Near the window was the work table with a microscope, a small microtome, and the most bizarre and disorderly population of objects, which made that table look more like that of a painter or a varnisher than that of a distinguished researcher. There were cardboard tablets holding preparations, slides of microscopic preparations […], bottles with liquids of various colors among which the chrome yellow of potassium dichromate prevailed, the blackish gray of silver nitrate, the dark green of copper acetate, and the blue green of sulfate; then a quantity of those characteristic jars of Liebig meat extract, blackened by silver nitrate, arranged in no particular order, which Golgi used for his black reaction; finally slips of paper with sketches of drawings” (Pensa 1991).

## Tendon sensitivity

While Golgi’s laboratory was emerging internationally in neuroanatomical and neurohistological studies and Golgi had the black reaction in his hands, he did not neglect to turn his attention to other fields of investigation as well. His intuition made him imagine that they could obtain important results.

The material basis of tendon sensitivity that underlay motor and proprioceptive mechanisms was a topic which generated conflicting views. Various physiological experiments had shown that anatomical knowledge of the relationships between nerves and tendons was incomplete.

Before Golgi, two researchers had tried to identify the material substrate of tendon sensitivity. Alexander Rollet (1834–1903), an Austrian physiologist, had studied the tendons of amphibians and found a plexus of myelinated fibers resulting from the division of the nerve in charge of it. From this plexus other fibers would originate and would further be divided in single “point” or with a final expansion in the end (Rollet 1876). Carl Sachs (1853–1878), who worked in the physiological laboratory of the University of Heidelberg, investigated the presence of nerve endings in the tendons of reptiles, amphibians, birds and mammals. He found constant endings in the tendons of amphibians and lizards where the nerve fiber eventually resolved into a brush of pale arborizations. In frogs he also observed a club-like termination. On the other hand, according to Sachs, such figures were missing in mammals where nerves would merge in the substance of the tendon, without specific morphological characteristics (Sachs 1875).

In Golgi’s research program was the idea that since there were nerve fibers within the tendons, following their course would lead to the discovery of their termination patterns.

Golgi’s originality was evidenced by his applying established histological techniques in a new context for this type of investigation.

**Fig. 2:**
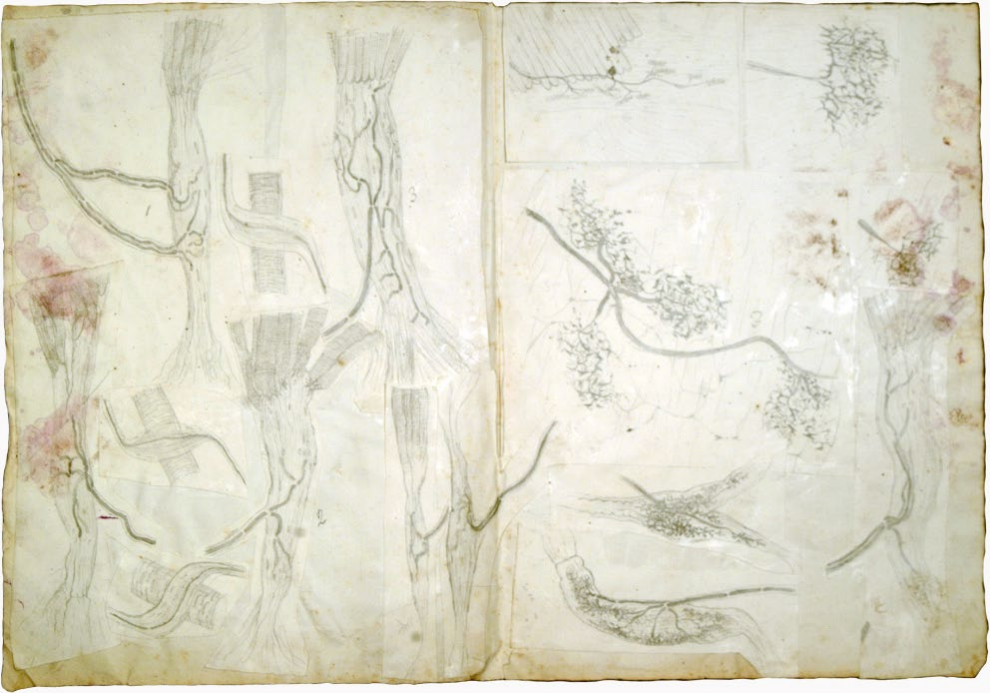
Pencil sketches (around 1878–1879) by Camillo Golgi of observations on sensory corpuscles in tendons. University of Pavia, Museum for the History of the University, Veratti Archives

The focus of his research was on human tendons. However, he also extended his “observations to several other mammals (rabbit, dog, cat, mouse), to some birds (sparrow, finch, swallow) and to amphibians (frog) and reptiles (lizard)” (Golgi 1880). Golgi declared that he was indeed guided - somewhat phylogenetically - to his results obtained in human anatomy by the observations he had made first in lizards, then in birds and finally in rabbits. The observation of nerve endings in reptiles, constituted almost a kind of cognitive map that made him clearly perceive the fundamental structure that he would later characterize also in mammals and in humans (Golgi 1880) (Fig. 2).

Golgi used several methods of histological procedures. After he carefully succeeded in separating the tendon from the muscle, he subjected the tendon to chemical treatment with solutions of certain acids (e.g., acetic, hydrochloric, or nitric acid) that had the effect of removing the natural opacity of the tendon that could hinder the observation of nerve fibers. To make the treatment more obvious, the tendon was also treated with osmic acid solutions that caused blackening of the nerve fibers themselves. These procedures also allowed the preparations to be preserved in glycerin. This first phase was followed by Golgi’s immersion in arsenic acid followed by a passage in a solution of gold chloride and potassium.

**Fig. 3:**
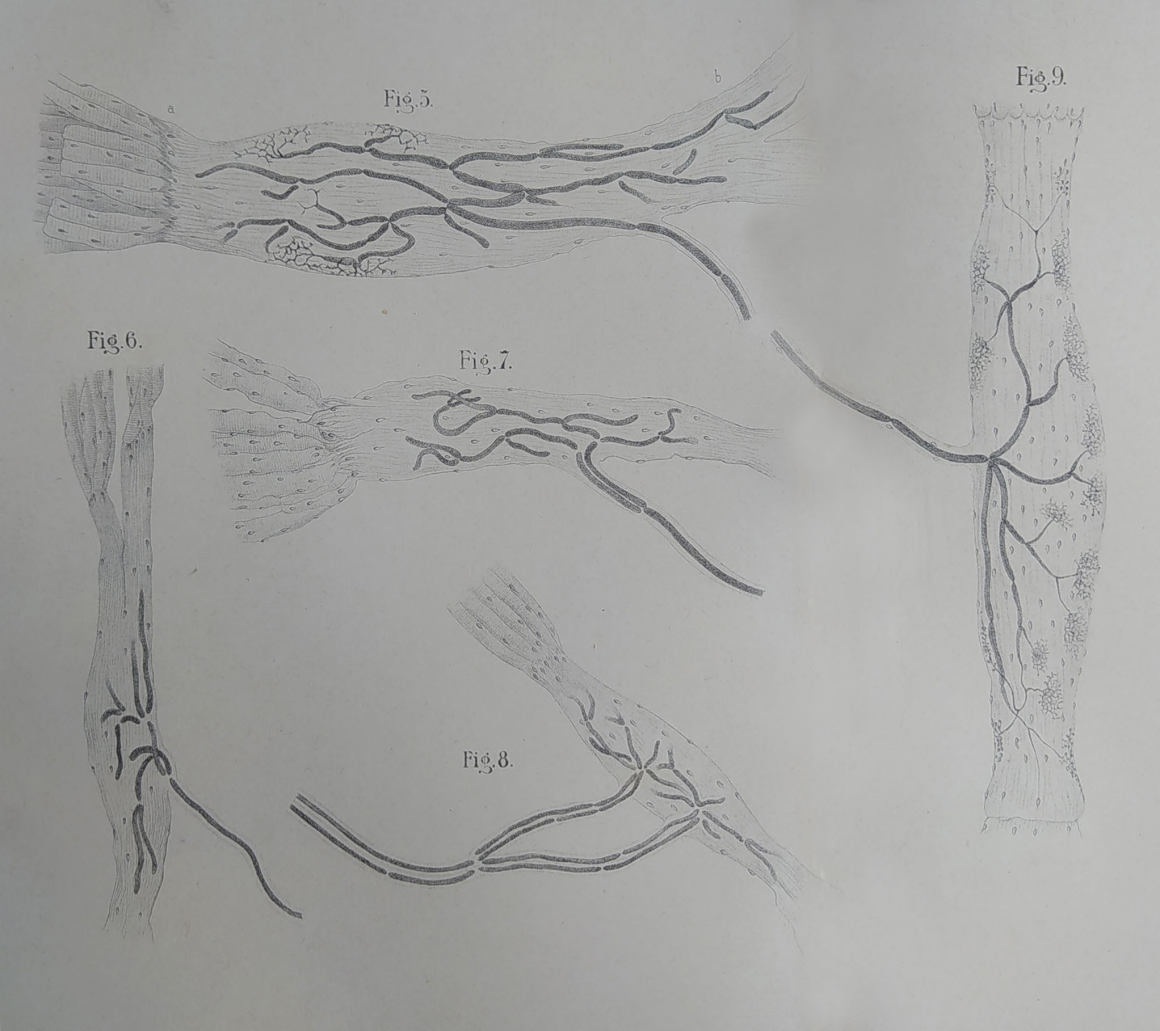
Golgi’s muscle-tendon organs. Originally published in Golgi in 1880 and reprinted in the Opera Omnia (Golgi 1903). University of Pavia, Camillo Golgi Museum

At the end of this procedure, microscopic observation made to highlight spindle formations possible that at one end appeared to stand near the tendon–muscle junction, almost in relation to muscle fibers, while at the other end they gradually blended with tendon bundles. Golgi discovered that each of these formations received from one to four myelinated nerve fibers, which lost their myelin sheath as they entered the bundle, within which they divided dichotically, terminating in a large number of ending arborizations that had the appearance of reticular intertwines (Fig. 3).

**Fig. 4:**
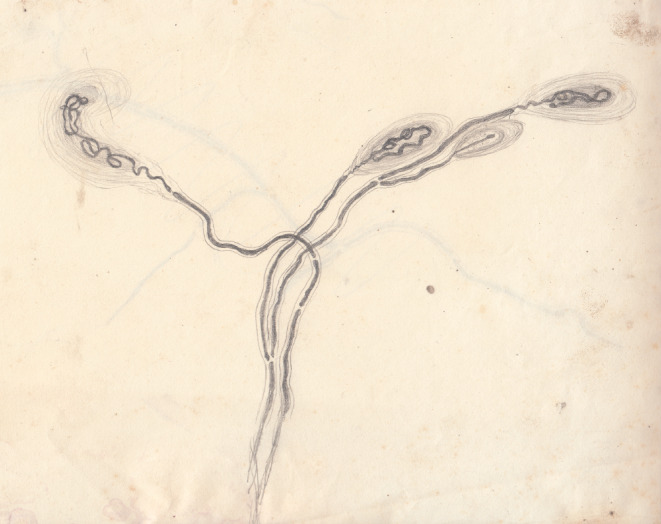
Pencil sketch (around 1878) of Pacini body-like tendon corpuscles (Golgi Mazzoni corpuscles). University of Pavia, Museum for the History of the University, Veratti Archives

He also observed, already at the first stage of the procedure, a second category of formations in the superficial thickness of the tendon, near the muscle (body like clubs and Pacini body-like formations) (Fig. 4).

Considering the position of the first corpuscles, which he called *muscle-tendinous organs*, Golgi concluded that they - given their position - had a “harmonizing function with that of the muscles” and, precisely, that they could “be organs of a special muscular sensitivity,” the “gauges of muscle tension” (Golgi 1880).

“As for the second type of terminal nervous apparatuses”, Golgi observed, “their more superficial situation” and their “analogy with other terminal organs of known function” led to the idea that they were “tactile bodies” (Golgi 1880), that is, those responsible for coding sensitivity to pressure (baresthesia).

**Fig. 5:**
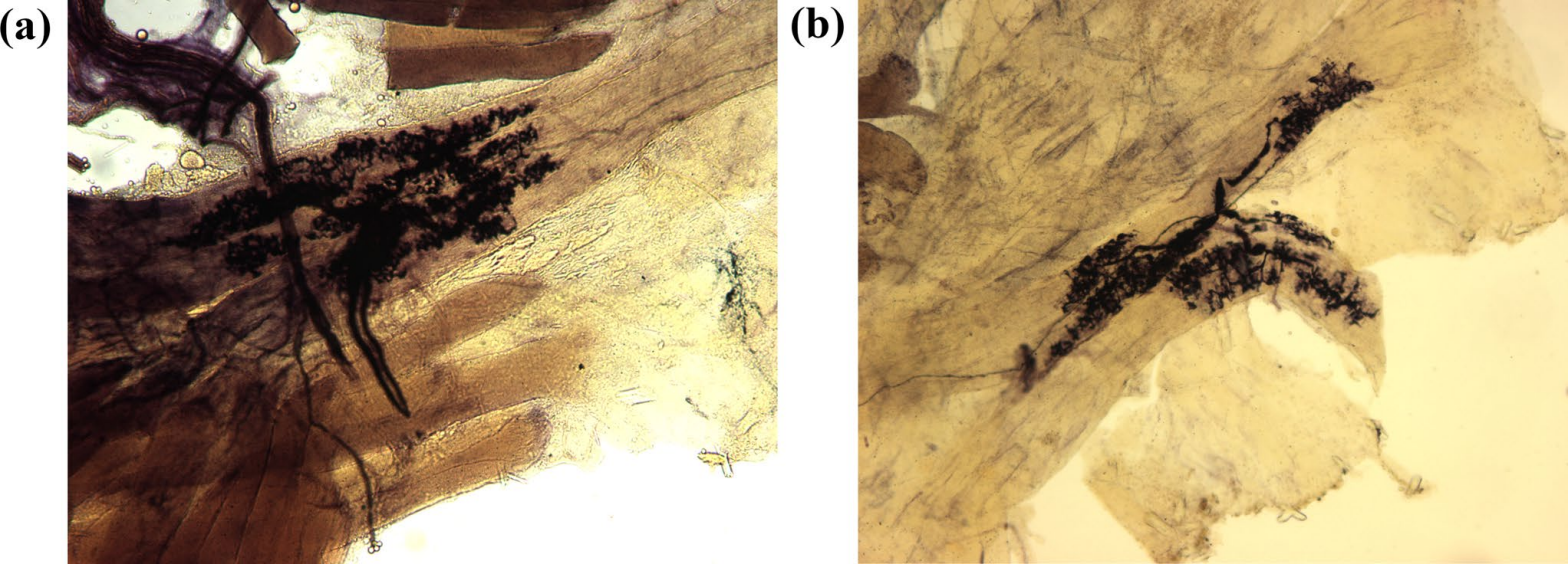
**(a**, ** b)** Golgi muscle-tendon organs. Photomicrograph (20x) from original preparations from the histotheca of Golgi’s laboratory (presumably dated around 1878–1880). University of Pavia, Camillo Golgi Museum

Thus Golgi, on the basis of shape, position and characteristics alone, had precisely and correctly identified the function of the sensory corpuscles had discovered. Golgi found it astonishing that “despite of the diligence with which anatomical research were conducted in the modern epoch” this discovery “had hitherto remained unnoticed by anatomists”: peculiarities of organization “so pronounced, so easy to prove and […] of such relevant physiological signification,” such as those underlying “the relation of nerves to tendons” (Golgi 1880) (Fig. 5a and b).

Golgi presented his research in 1878 with a preliminary note (Golgi 1878a), and soon afterwards with another brief communication to the seventh extraordinary meeting of the Italian Society of Natural Sciences (Golgi 1878b). To conclude this type of research he published a detailed work in the proceedings of the Royal Academy of Sciences of Turin (Golgi 1880).

Preliminary notes were immediately reviewed in the influential German medical journal *Centralblatt für die medicinischen Wissenschaften* (1876) and taken up by the histologist Ludwig Thanhoffer (1843–1909) - professor in Budapest - in a communication to the Hungarian Academy of Sciences (Thanhoffer 1882). The discovery was soon described even in elementary histology textbooks (Klein 1885, pp. 209–210).

Golgi’s name, as the eponym for the muscle-tendon, organs entered the scientific literature almost immediately thanks to the work by Vittorio Marchi, a young student who practiced in the Ophthalmology clinic of the University of Pavia, and who later became internationally famous for developing a method of staining central nerve pathways. Marchi succeeded in demonstrating the existence of Golgi muscle-tendon organs in the tendons of the striated muscles of the eye (where Golgi had failed to find them), calling them *Golgi bodies* (Marchi 1882). Alfonso Cattaneo (second half of the 19th century) also studied these corpuscles in a very detailed investigation under pathological conditions. After cutting spinal nerves he observed a degeneration of peripheral nerve fibers and some wrinkling of the fusiform structure of the organ (Cattaneo 1888a, b).

The naturalist Sergio Pansini named the muscle-tendon organ with the term Golgi corpuscles in a work of his (Pansini 1888).

In 1889, the authoritative *Handbuch der Gewebelehre des Menschen* by Albert von Kölliker (1817–1905) called the muscle-tendon organs as *Golgi’sche Sehnenspindeln* (*Golgi’s tendon spindles*), in honor of their discoverer (Kölliker 1889) but also *Golgi’s organs*, a term also taken up in an important Italian treatise on histology (Fusari and Monti 1891); the eponym *Golgi’s muscle-tendon organs* was also used (Ciaccio 1889; Ruffini 1894). The name *Golgi tendon organ(s)* came into common use during the twentieth century (Moore 1984; Jami 1992).

A real advance in the analysis of nerve endings in tendons was achieved with the study by Vittorio Mazzoni (1864–1897) devoted to the formations already observed by Golgi who had described them as “balls, clubs and different forms of Pacinian corpuscles” (Golgi 1880; Mazzoni 1890; Arieti 2008). This study, which defined more precisely the morphology of this second category of tendon nerve endings, was taken up again by Angelo Ruffini (1864–1929) who proposed to call these formations *Golgi-Mazzoni corpuscles*, from the name of their discoverer and of the person who had described their morphological aspect with precision (Ruffini 1894).

During the twentieth century, studies on the two sensitive corpuscles discovered by Golgi flourished with the publication of hundreds of articles, especially on the Golgi tendon organ. Several researches have studied Golgi tendon organ structure (Nitatori 1988; Rein et al. 2023), its physiology (Houk and Simon 1967; Kistemaker et al. 2013; Oliver et al. 2021) and functions in various conditions of mechanical stress (Gregory et al. 2003; Qiu and Kang 2017), its role in sports activity (Moore 2007) and its involvement in pathological conditions (Burne and Lippold 1996). More limited research has been done on the Golgi-Mazzoni corpuscles which are slowly adapting receptors sensitive to particular types of compression in the joint capsule (Grigg et al. 1982; Chouchkov and Maslarski 2019).

## Conclusions

With the discovery of the muscle-tendon organs and the observation of what were later called Golgi-Mazzoni corpuscles, Camillo Golgi made a major contribution to microscopic anatomy, histology and human physiology. In fact, he recognized their morphological characteristics and was able to hypothesize their precise physiological meaning. These types of sensitive endings are also able to contribute to the perception of the body’s position in space and are, therefore, also fundamental for proprioceptive sensitivity.

## Data Availability

All texts cited in the article can be found through the use of public libraries. Archival documents can be consulted upon request to be submitted to the Museum for the History of the University of Pavia (https://musei.unipv.eu/msu/il-museo/biblioteca-e-archivio/) Observation of the slides can be obtained from the Camillo Golgi Museum (https://musei.unipv.eu/msu/il-museo/biblioteca-e-archivio/)
